# Targeting Glioma with a Dual Mode Optical and Paramagnetic Nanoprobe across the Blood-brain Tumor Barrier

**DOI:** 10.4172/2157-7439.1000395

**Published:** 2016-08-31

**Authors:** Kishor Karki, James R Ewing, Meser M Ali

**Affiliations:** Department of Neurology, Henry Ford Hospital, Detroit, MI 48202, USA

**Keywords:** Glioma, Tumor blood-brain barrier, Dual modality, MRI, Optical imaging

## Abstract

In brain tumors, delivering nanoparticles across the blood-tumor barrier presents major hurdles. A clinically relevant MRI contrast agent, GdDOTA and a near-infrared (NIR) fluorescent dye, DL680 were conjugated to a G5 PAMAM dendrimer, thus producing a dual-mode MRI and NIR imaging agent. Systemic delivery of the subsequent nano-sized agent demonstrated glioma-specific accumulation, probably due to the enhanced permeability and retention effect. *In vivo* MRI detected the agent in glioma tissue, but not in normal contralateral tissue; this observation was validated with *in vivo* and *ex vivo* fluorescence imaging. A biodistribution study showed the agent to have accumulated in the glioma tumor and the liver, the latter being the excretion path for a G5 dendrimer-based agent.

## Introduction

Gliomas (GBMs) present great difficulties in treatment; even best practice in the age of temozolomid yields median survival times of about 15 months [1]. Standard practice – surgery and radiation therapy followed by adjuvant chemotherapy - very often fails, both because of uncertainty in delineating the margin of the tumor [2] and because of the infiltrative nature of glioblastoma (GBM), making it essentially impossible to resect the entire tumor mass [3–5].

As for chemotherapeutic approaches, the delivery of anti-cancer agents to brain tumors such as GBMs represents a challenge because the blood-brain barrier (BBB) effectively limits the delivery of many agents, and the high tumor interstitial fluid pressure presents an additional delivery barrier [6]. However, nanotechnology has demonstrated the potential to transfer drugs across the BBB [7] and into brain tumors [8].

In order to meet their very high metabolic demands, brain tumors exhibit high levels of angiogenic activity, albeit the subsequent vasculature is disorganized, with tortuous and abnormally dilated vessels, leaky inter-endothelial gaps, fenestration, and the absence of perivascular elements that generate the BBB in normal tissue [9–11]. This hyper-permeable vasculature and deficient drainage allow nanoparticles to extravasate and be retained in tumor interstitium following systemic administration - a phenomenon referred to as the Enhanced Permeability and Retention (EPR) effect [12]. Although there is no lymphatic system in the brain, clearance of the interstitial fluid by bulk flow through the white and gray matter is comparable to the average lymph flow rate in other tissues; as result, the EPR effect of carriers for peripheral solid tumors has also been observed in brain tumor [6,11]. Nonetheless, effective transvascular delivery of nanoparticles across the blood-brain tumor barrier (BBTB) of malignant gliomas remains a challenge, partly because of our limited understanding of nanoparticle properties in relation to the physiologic size of pores within the BBTB.

Nanoparticle-based drug delivery cannot be expected to be equally effective across tumor types, sizes, locations, stages and grades. The properties of dendrimer-based Gd-DTPA agents *in vivo* depends on the size, core and the exterior surface charge [13,14] and the porosity and pore size of tumor vessels vary with the type and status of the tumor. In a recent study a series of PAMAM dendrimer-based Gn-Gd-DTPA (G1 to G8) were synthesized and the pharmacokinetics of the synthesized agents were studied in the BBTB of glioma tumor bearing rats [12,15,16]. It was demonstrated that gadolinium chelated dendrimer nanoparticles with core sizes of <12 nm permeated the BBTB, whereas larger nanoparticles were hindered [17]; thus, the upper limit of pore size in the BBTB of malignant brain tumors is approximately 12 nm [12,18,19]. Spherical dendrimer-based paramagnetic nanoparticles ranging between 4 to 10 nm in diameter maintain peak blood concentrations for several hours [6,12,17].

One reservation about previous work is that the ligand motif used to bind Gd^3+^ build the dendrimer-based nanostructures was DTPA, a less thermodynamically stable linear acyclic ligand than macrocyclic chelators. In an attempt to prepare clinical relevant MRI contrast agent for glioma imaging, we synthesized a generation 5 (G5) dendrimer conjugated with thermodynamically stable macrocyclic Gd-DOTA chelates. Finally, in order to address the relatively low T1 relaxivity of Gd^3+^ at high fields, a dual-mode approach was adopted to solve this problem, incorporating a fluorophore into the MRI contrast agent thus producing a more sensitive probe for location of the sites of nanoparticles in tissue.

A dual mode MRI-optical approach is ideally suited for *in vivo* biomedical imaging because MRI provides noninvasive *in vivo* high resolution anatomical images, while fluorescence imaging has high sensitivity and can provide microscopic information in postmortem pathological tissues. Although various types of fluorescent dyes have been conjugated with MRI contrast agents, their half-lives were too short for longitudinal *in vivo* studies [2,20]. Small molecule fluorescent dyes such as such as rhodamine [21], fluorescein [22], napthalimide [23,24] and BODIPY [25] have been widely used to design a dual mode probe for biomedical applications. However, these probes emit at the visible region of the spectrum which is not favorable for *in vivo* imaging, since visible light penetration in in tissue is limited to about 1 cm depth, thus limiting applications of optical imaging to skin cancer or endoscopy. On the other hand, near infrared (NIR) dyes have better tissue penetration properties [20]. This is particularly important in brain tumors, where light penetration is more difficult due to bone-enclosed structures. To our knowledge, the development of an NIR-T1 relaxation based MRI probe for *in vivo* imaging of glioma is unique to our laboratory.

In our previous report, we conjugated a NIR dye, DyLight680 (DL680) with dendritic PARACEST (Paramagnetic Chemical Exchange Saturation Transfer) agent to detect glioma *in vivo* [11]. A dendrimer-based paraCEST-NIR agent was delivered to glioma in a compromised BBTB. Keeping this in mind, we developed a dendrimer-based dual mode probe incorporating more clinically applicable Gd-DOTA in combination with an NIR dye, DL680 with the potential application in glioma imaging with a compromised BBTB.

## Materials and Methods

All commercially available reagents were purchased from Sigma-Aldrich, and were used as received unless otherwise noted. The ethylene diamine core PAMAM G5 dendrimer with primary amines on its surface was purchased as 20 wt% solution in methanol from Dendritech Inc. (Midland, MI). The ligand S-2-(4- Isothiocyanatobenzyl)-1,4,7,10-tetraazacyclododecane-tetraacetic acid (p-SCN-Bz-DOTA) was purchased from Macrocyclics, Inc. (Dallas, TX). Ultrafiltration membranes (Amicon-Ultra MWCO 30 kDa) were obtained from Millipore (Billerica, MA). Dendrimeric chelates and their conjugates were purified by repeated ultrafiltration with deionized water using appropriate molecular weight cut-off Millipore’s Amicon Ultra centrifugal filters. Matrix-assisted laser desorption/ ionization time-of-flight (MALDI-TOF) mass spectra were acquired on an Applied Biosystems Voyager DE spectrometer at Scripps Center for mass spectrometry. The Gd^3+^ content was measured by inductively coupled plasma-mass spectroscopy (ICP-MS) at Chemical Solutions Ltd. The relaxation times of ( GdDOTA)_54_-G5 PAMAM were measured on a horizontal bore 7 T Varian/Agilent MRI system (Palo Alto, CA) equipped with a 20 mm^1^H surface coil (Rapid MRI, Columbus OH). All MR experiments were conducted at temperature of 35°C [11,26].

### Tumor implantation and MRI methods

All animal experiments were approved by Institutional Animals Care and User Committee (IACUC). Orthotopic human glioma was created in nude rats by implanting 4 x10^5^ human U251 glioma cells according to our recent published method [27–29]. Animals were killed under deep anesthesia, and brain tissues were collected after perfusion using ice-cold 1 x PBS.

High-resolution T1-weighted images were acquired pre- and post-administration of the (GdDOTA)_54_-G5-DL680 contrast agent with the following parameters: matrix: 256 x 192, 27 slices, 0.5 mm thickness, no gap, NE=1, NA=4, TE/TR=16/800 ms. Prior to the administration of contrast agent, two Look-Locker (LL) sequences were run so that a voxel-by-voxel estimate of T_1_ in the tissue could be made. A solution of 0.05 mmolGd/kg of (GdDOTA)_54_-G5-DL680 (14 mM in 600 μL PBS) was injected through the tail vein prepended by 100 μL of heparinized saline and followed by 100 μL of saline to flush the catheter. The total volume of 800 μL was manually injected in 1 min. Immediately after the administration of contrast agent, an additional 40 LL studies were run at intervals of approximately 136 s. LL sequence parameters were as follows: matrix: 128 x 64, five 2.0 mm slices, no gap. NE=24 inversion-recovery echoes, TR=2000 ms. The total time of study after CA administration was approximately 92 minutes.

### Optical imaging

After acquisition of MR images, near-IR optical imaging of the rat was performed using a Kodak Carestream Multispectral Imaging System (Carestream, USA). Fluorescence images were acquired using multiple excitation wavelength filters ranging from 540 to 690 nm and a single emission wavelength filter of 750 nm. Spectral profiles were created using spectral analysis software (Carestream) and subtracted optimal images were obtained. For each optical image set, an X-ray image was also obtained to validate the anatomic location of the tumor as well as major organs (the Fluorescence and X-ray images were overlaid). To validate dual mode *in vivo* imaging of glioma, the rat was sacrificed in an appropriate state of anesthesia [11] in the next day and the major organs were collected after perfusion for ex-vivo fluorescence imaging. The brain was snap-frozen and cut into 20-40 μm thick sections for further analysis with fluorescence microscopy. For fluorescent microscopic detection of DL680 conjugated to G5, a proper excitation and emission filter was used.

### Transmission electron microscope (TEM) and zeta potential measurement

To determine the size of nanoparticles TEM was carried out using a JEOL 2010 microscope operating at 200 kV. Samples were prepared by diluting the nanoparticle solution and depositing a drop on a copper grid coated by a thin film of amorphous carbon, allowing the liquid to dry in air at room temperature. The exterior surface charge of (GdDOTA)_54_-G5-DL680 particle was assessed by zeta potential measurement (Malvern zetasizer nano series).

#### Synthesis of (DOTA)_54_-G5-PAMAM

The synthesize of DOTA-G5 PAMAM dendrimer was conducted by following our recent published method, except that G5-PAMAM dendrimer (40 mg, 1.4 μmol) and p-SCN-Bz-DOTA (150.0 mg, 218 μmol) were used. The final conjugate was purified by diafiltration using a Centricon C-30 cell with a 30 kDa molecular weight cut-off, after which the solvents were removed by lyophilization to afford a colorless solid (0.22 g).

#### Preparation of Gd^3+^ Complexation

To synthesize paramagnetic dendrimeric chelates, Gd-DOTA)_54_-G5, 1.2 equivalent of GdCl_3_ in H_2_O was added to a solution of DOTA-G5 (0.050 g) in H_2_O (pH 6–7). The reagents were stirred at 40°C for 48 h before EDTA was added to remove free metal ions. The final solutions were repeatedly filtered through Amicon Ultra 10 kDa MWCO filter. The filtered solutions were examined by xylenol orange test to confirm no free metal ions were present. Following purification, the final dendritic conjugate was characterized by MALDI-TOF. The average molecular weight of Gd^3+^chelated dendritric conjugate was estimated at 66,914 g/mole (Figure S1). This corresponds to a G5-dendrime with an average of 54 chelated Gd^3+^ ions per dendrimer. This mass spectrum also showed a low, broad feature at approximately twice the m/z ratio of the main peak, which indicated a low level of dimerization of some dendrimers.

#### Synthesis of (GdDOTA)_54_-G5-DL680

To synthesize of (GdDOTA)_54_-G5-DL680, a solution of DL680-NHS (0.067 g, 127 μmol) in dimethyl sulfoxide was added to a stirred solution of (GdDOTA)_54_-G5 (40 mg, 0.60 μmol) in 2 ml of PBS (pH 7.5), and the reaction was stirred at room temperature for 12 h. The reaction mixture was diafiltered using Amicon Ultra centrifugal filter unit with a 10 kDa molecular weight cut-off at 3k rpm to remove hydrolytic by-products. The solution was lyophilized to obtain 30.2 mg of solid conjugate. The labeling degree of rhodamine was determined by measuring the absorbance of DL680 (ε_692_=140,000 M^−1^cm^−1^), and 1.1 molecules of DL680 were conjugated with each dendrimer molecule.

## Results and Discussion

**Figure 1:**
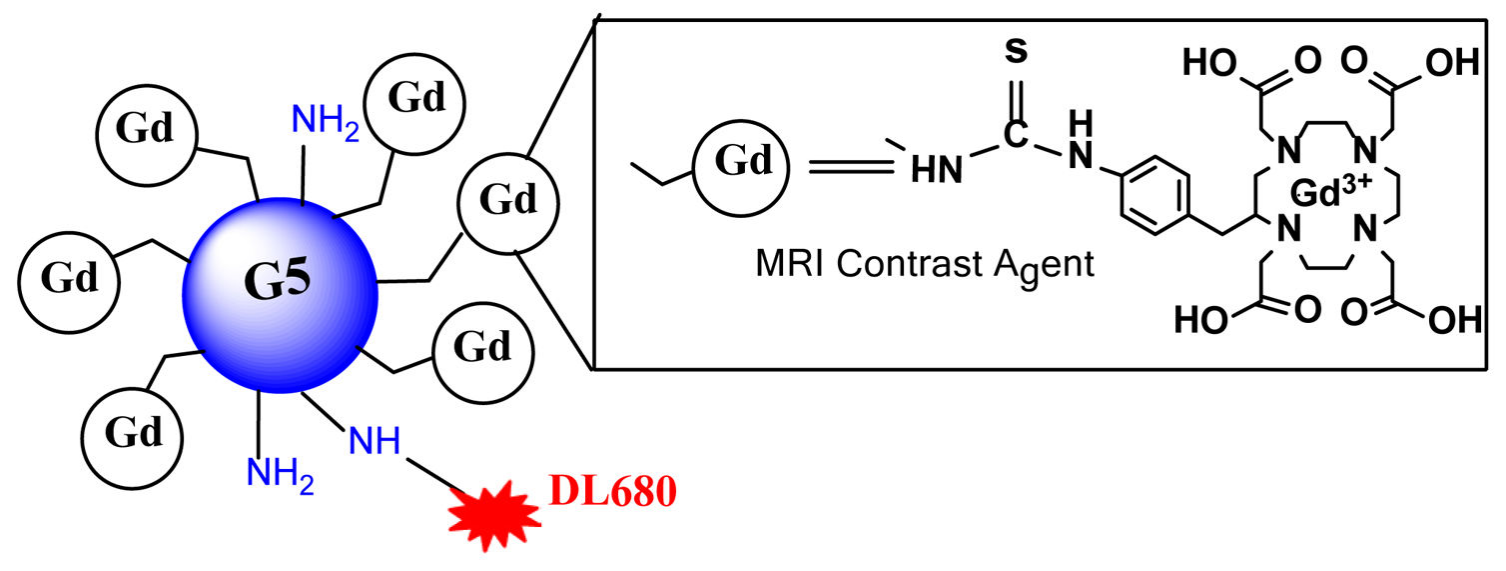
Schematic view of Gd^3+^ chelated with 1,4,7,10-tetraazacyclododecane- 1,4,7,10-(DOTA) in a G5 PAMAM dendrimer with DyLight (DL680).

**Figure 2:**
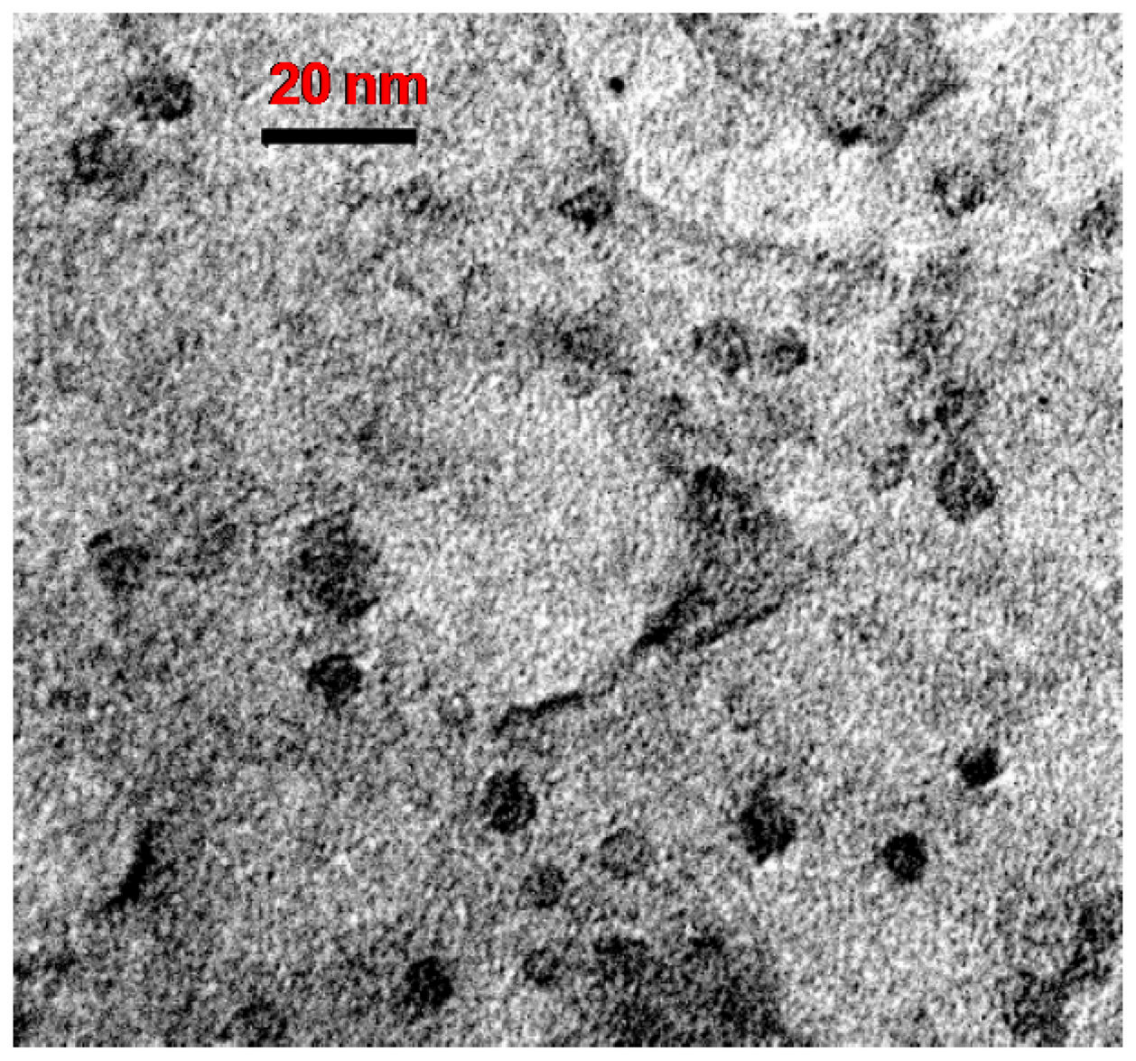
TEM image of (GdDOTA)_54_-G5-DL680 (schematic view is shown in Figure 1). TEM image shows a uniform distribution of the Gd-G5-DL680 nanoparticles. The particles are rounded with an average size of 7.6 ± 0.6 nm (scale bar=20 nm).

In our recent report [29], p-SCN-Bz-DOTA was conjugated to the amines on the surface of PAMAM dendrimer from generation 1 (G1) to generation 4 (G4) PAMAM dendrimer by using isothiocyanatobenzyl group. Here, that study is extended by conjugating p-SCN-Bz-DOTA to the amines on the surface of a generation 5 (G5) PAMAM dendrimer, achieving excellent synthesis yield and purities (Figure 1). Following this, the chelation of Gd^3+^ ion for G5 dendrimeric conjugate was conducted. MALDI-TOF mass spectroscopy revealed an average of 54-GdDOTA molecules conjugated with a generation G5 PAMAM dendrimer (Figure S1). The size of Gd_54_-G5-DL680 dendrimer was estimated by TEM to be approximately 7.6 ± 0.6 nm (Figure 2). The exterior surface charge of this particle was assessed by zeta potential measurement to be 2.1 ± 1.2 mV at pH 7.4.

**Figure 3:**
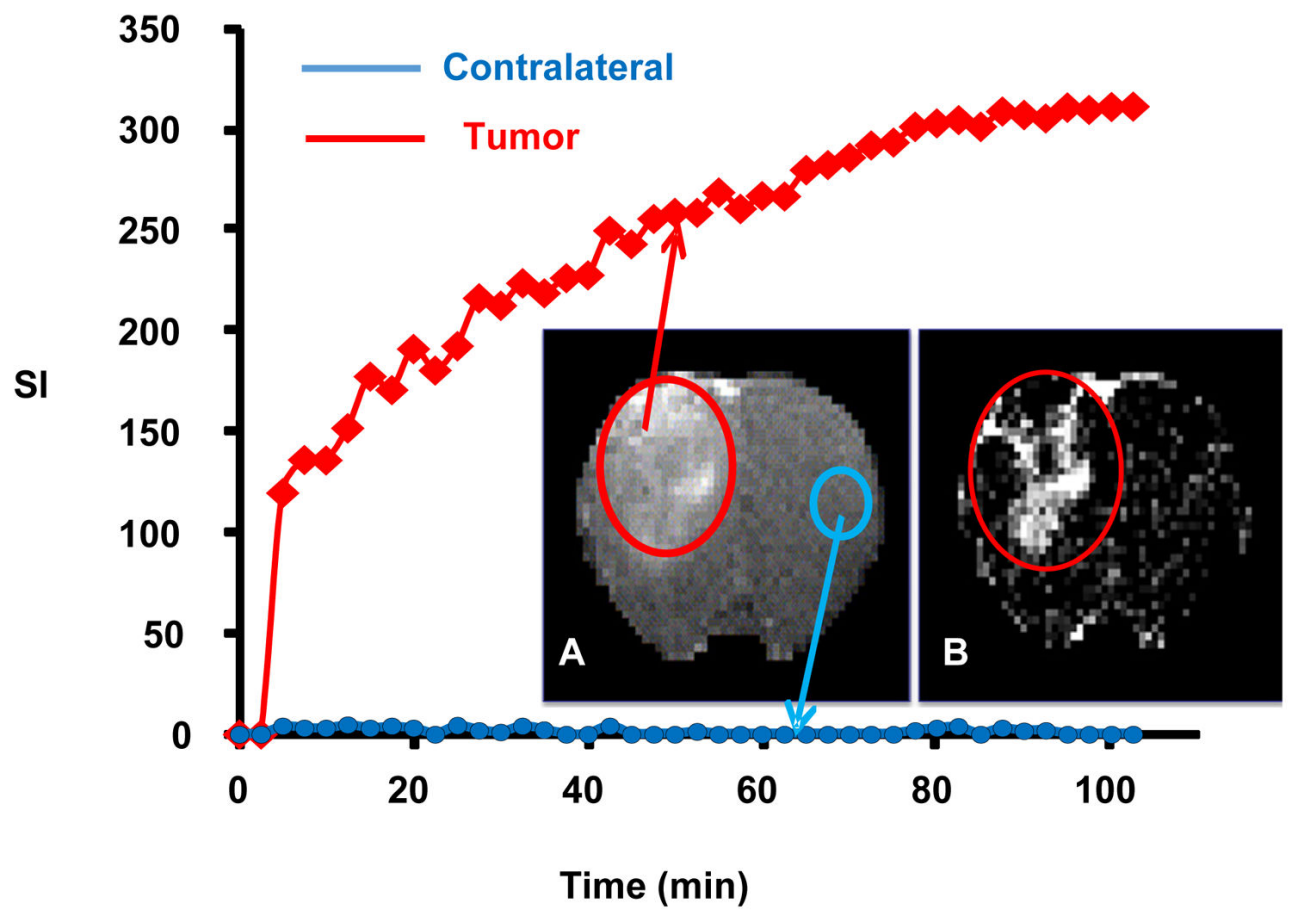
(A) T1-weighted image (last image of LL sequence acquisition) showing the presence of (GdDOTA)_54_-G5-DL680 nanoparticle within U251 glioma bearing rat. (B) Subtraction image, one-hour post-contrast signal intensity minus pre contrast signal intensity. In the accompanying graph, the red trace tracks the accumulation of contrast in the tumor (red ellipse), while the blue trace tracks contrast in the contralateral side of normal contralateral brain (blue ellipse). The graph clearly demonstrates that agent extravasates across the BBTB and accumulates over time within extravascular space of tumor tissue, but there is no such accumulation of the agent at the contralateral site of the normal brain.

**Figure 4:**
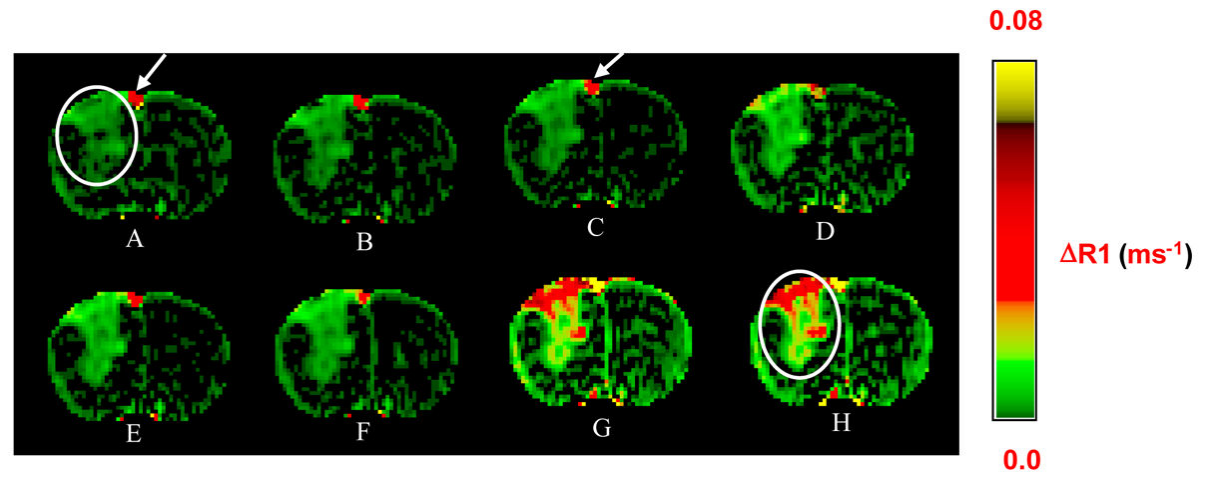
Pixel-by-pixel ΔR1-maps in a U251 glioma tumor. ΔR1-maps are presented at different time points post contrast administration: 12 min (A), 24 min (B), 36 min (C), 48 min (D), 60 min (E), 72 min (F), 84 min (G) and 96 min (H). The injected agent was Gd-G5-DL680. ΔR1-maps clearly demonstrate accumulation of the agent concentration within tumor over time. Tumor is indicated as white circle. Arrows indicate sagittal sinus.

The pharmacokinetics of (GdDOTA)_54_-G5-DL680 was studied by *in vivo* MRI. Systemic delivery of (GdDOTA)_54_-G5-DL680 led to the visualization of the agent in the rat glioma tumor over the course of 92 min post-contrast administration. Figure 3 shows, in a U251 glioma, the time trace of increase in signal intensity in the last echo of each of the 40 LL image sets. Figure 4 shows pixel-by-pixel R1 maps at different time points in tumor and normal tissue, ΔR1=R1post - R1pre, where R1post and R1pre are the pre- and post-contrast relaxation rates, respectively, and R1=1/T1.

Both Figures 3 and 4 show that nanoparticle concentration in the tumor was increasing over time. This observation is consistent with lipid-insoluble macromolecule maintaining peak blood concentrations for several hours (6), since macromolecules 7 nm and larger are not cleared by the kidneys [30].

**Figure 5:**
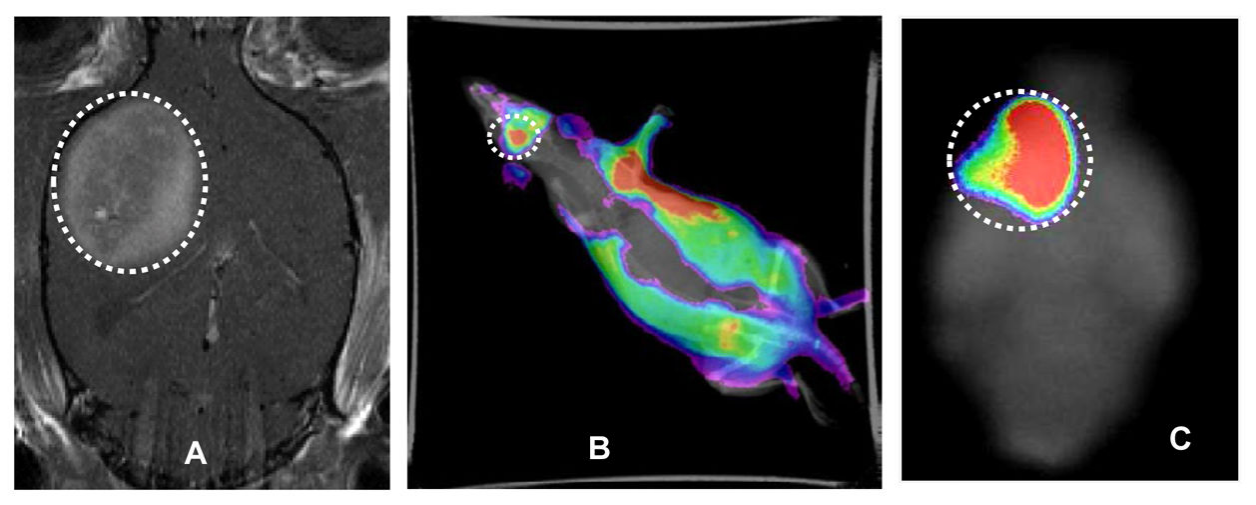
The coronal *in vivo* MRI image shows the location of U-251 glioma tumor (A). The agent was (GdDOTA)_54_-G5-DL680 and injected at a dose of 0.04 mmolGd/kg. *In vivo* optical image obtained under simultaneous white light and filtered (540-690 nm) excitation detected with the emission filter set at 750 nm demonstrating fluorescence in the glioma (B). Ex-vivo fluorescence imaging of rat brain clearly shows the selective accumulation of the Gd-G5- DL680 within the tumor (C): Tumors are indicated as dotted white circles.

**Figure 6:**
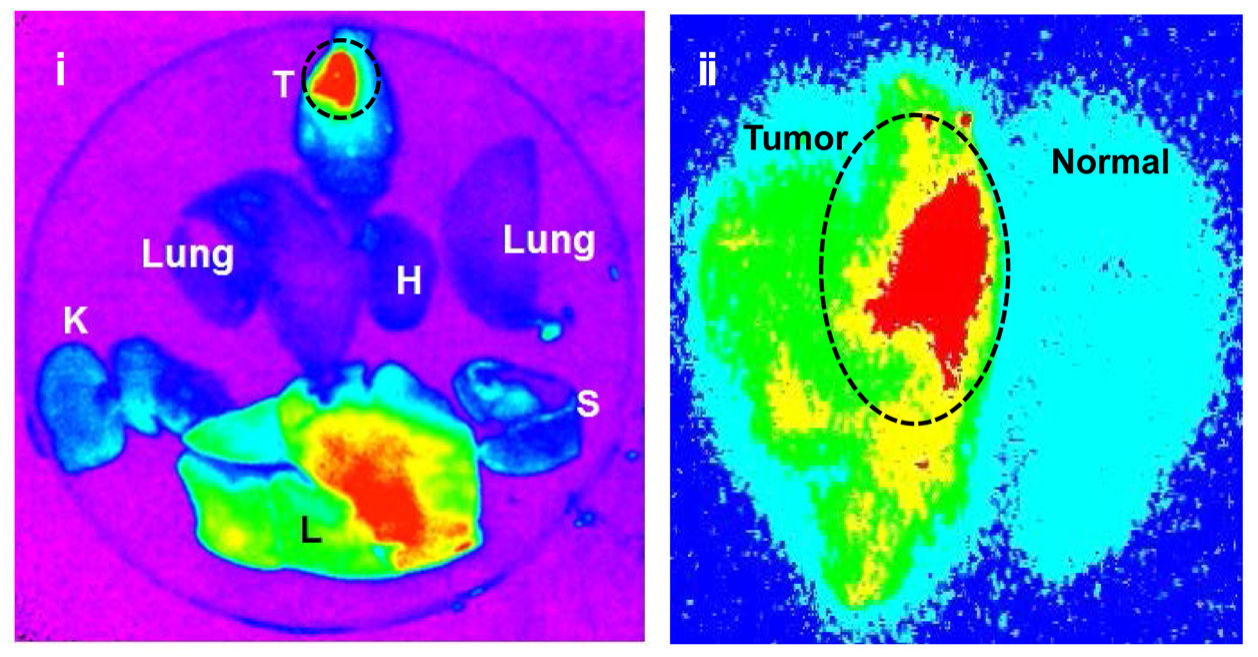
Distribution of (GdDOTA)_54_-G5-DL680 in organs: (i) NIR fluorescence images of brain (T), kidney (K), spleen (S), liver (L), heart (H) and lung. (ii) *Ex vivo* fluorescence imaging of a rat brain section (40 micron thick frozen brain tissue section) clearly showed the accumulation of the nanoparticles in the tumor selectively. Non-specific accumulation of the particles was not observed in the normal contralateral brain, is consistent with the contrast accumulation in the MRI images in Figures 3 and 4.

To take the advantage for dual mode nanoprobe, a coronal *in vivo* MRI image of a different rat with a U-251 glioma (Figure 5A) was acquired. Immediately after MRI acquisition, optical imaging of the same rat was performed, thus generating matched sets of dual modality imaging data of the tumor. Visualization of glioma tumor in the whole body fluorescence image is demonstrated in Figure 5B. We also observed fluorescence signal in organs other than the rat brain. After sacrifice, a high fluorescence signal was detected in the tumor (Figure 5C) and the liver (Figure 6i). Kobayashi et al. reported the excretion of a GdDTPA-G5 dendrimer through liver [31]. Thus, nanoparticles showed more accumulation in tumor by EPR effect and excreted through liver possibly as a result of small particle size, relatively neutral surface charge and hydrophilicity in blood. Another group has also reported that the bio-elimination of lipid insoluble macromolecules 7 nm and larger in size predominantly proceeds through liver clearance [30].

After the brain was snap-frozen it was cut into 20 to 40 μm thick sections for further *ex vivo* fluorescence imaging analysis. The specificity of *in vivo* MRI was further validated with *ex vivo* fluorescence imaging, which showed that the nanoparticle accumulated at the glioma tumor selectively, but not in the contralateral brain tissue (Figure 6ii). Furthermore, biodistribution of the agent revealed that the agent was accumulated into glioma tumor by EPR effect, and excreted through the liver. It appears that the agent has minimum systemic toxic effect to the major organs (Figure 6i).

Conjugation of Gd^3+^ chelates on the surface of dendrimers slows down the rotational molecular motion of the complexes, and thus increases their relaxivity. Kobayashi et al. have developed many dendrimer-based macromolecular contrast agents by attaching Gd^3+^ chelates such as Gd-DTPA to the periphery of the dendrimer [13,31,32]. These contrast agents have shown a higher relaxivity profile at clinical magnetic field strengths up to 3 T. Following recently published methods [26], the relaxivity of Gd_54_-G5-DL680 was determined at 7 T to be 5.67 mM^−1^s^−1^. This value is significantly lower than the relaxivity of a G5 dendrimer-based contrast agent at 3 T field strength [26,32], which is not surprising, since it is well known that the relaxivity of dendrimeric contrast agents is diminished as field increases to, in this case 7 T. To improve the utility of the agent for detection of solid tumors with leaky vasculature, we conjugated a DyLight680 to the (GdDOTA)_54_-G5 yielding a dual fluorescent and MR imaging probe (i.e., (GdDOTA)_54_-G5-DL680) which can be used for both MRI and optical imaging.

## Conclusion

We have developed a nano-sized dual mode imaging agent that used a G5 PAMAM dendrimer to carry clinically relevant Gd-DOTA and a fluorescent dye. Systemic delivery of (GdDOTA)_54_-G5-DL680 resulted in the agent homing into its glioma tumor site selectively. *In vivo* MRI detected the agent in a glioma tumor, but not in contralateral tissue; the specificity of the agent was validated by whole body NIR-optical imaging and *ex vivo* fluorescence imaging. The *in vivo* MRI showed the macroscopic location of the tumor while fluorescence imaging showed the biodistribution of the agent, demonstrating that dual-mode imaging agent has utility for practical applications.

## Supplementary Material

Supple file
