# Supplementary material for: Targeting Glioma with a Dual Mode Optical and Paramagnetic Nanoprobe across the Blood-brain Tumor Barrier
Source: J Nanomed Nanotechnol. Author manuscript; Available in PMC 2016 Sep 29. (PMC5042151; doi:10.4172/2157-7439.1000395)
Supplement: Supple file [file NIHMS817888-supplement-Supple_file.pdf]

Targeting Glioma with a Dual Mode Optical and Paramagnetic Nanoprobe across the Blood Brain Tumor Barrier

Kishor Karki[†], James R. Ewing[†] and Meser M. Ali[†]*

Supplementary materials:

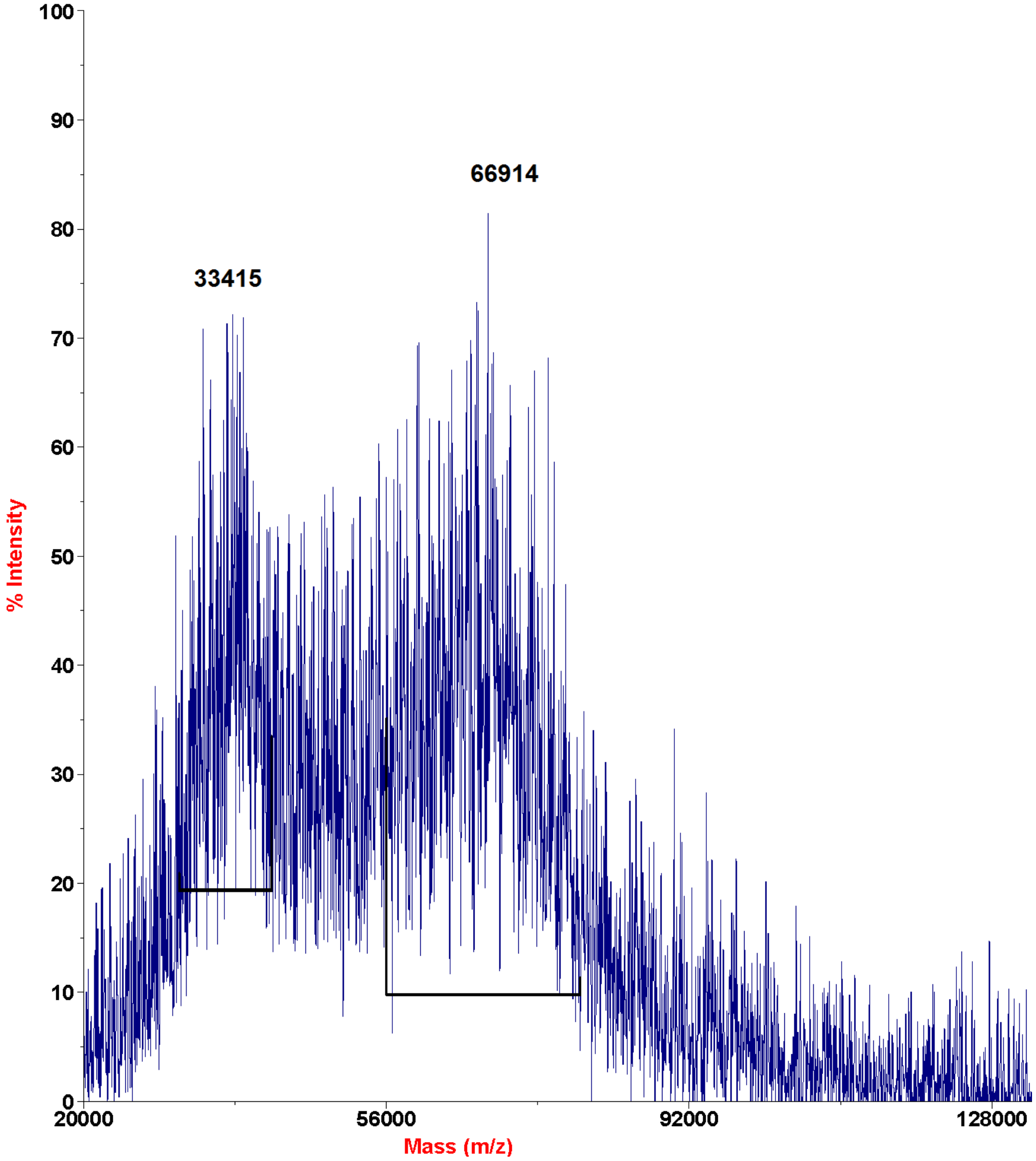

Figure S1: MALDI-TOF spectrum for molecular weight determination of **(GdDOTA)$_{54}$-G5**. The average number of **Gd-DOTA** conjugated with a G5 PAMAM dendrimer was estimated to be 54 per dendrimer.
